# JWA Deficiency Suppresses Dimethylbenz[a]Anthracene-Phorbol Ester Induced Skin Papillomas via Inactivation of MAPK Pathway in Mice

**DOI:** 10.1371/journal.pone.0034154

**Published:** 2012-03-26

**Authors:** Zhenghua Gong, Yaowei Shi, Ze Zhu, Xuan Li, Yang Ye, Jianbing Zhang, Aiping Li, Gang Li, Jianwei Zhou

**Affiliations:** 1 Department of Molecular Cell Biology & Toxicology, the Key Laboratory of Modern Toxicology, Ministry of Education and Department of Occupational Medicine and Environmental Health, School of Public Health; Nanjing Medical University, Nanjing, People's Republic of China; 2 Jiangsu Key Lab of Cancer Biomarkers, Prevention and Treatment, Cancer Center; Nanjing Medical University, Nanjing, People's Republic of China; 3 Department of Dermatology and Skin Science, Jack Bell Research Centre, Vancouver Coastal Health Research Institute, University of British Columbia, Vancouver, British Columbia, Canada; University of Florida, United States of America

## Abstract

Our previous studies indicated that JWA plays an important role in DNA damage repair, cell migration, and regulation of MAPKs. In this study, we investigated the role of JWA in chemical carcinogenesis using conditional JWA knockout (*JWA* ^Δ*2/*Δ*2*^) mice and two-stage model of skin carcinogenesis. Our results indicated that *JWA* ^Δ*2/*Δ*2*^ mice were resistant to the development of skin papillomas initiated by 7, 12-dimethylbenz(a)anthracene (DMBA) followed by promotion with 12-O-tetradecanoylphorbol-13-acetate (TPA). In *JWA* ^Δ*2/*Δ*2*^ mice, the induction of papilloma was delayed, and the tumor number and size were reduced. In primary keratinocytes from *JWA* ^Δ*2/*Δ*2*^ mice, DMBA exposure induced more intensive DNA damage, while TPA-promoted cell proliferation was reduced. The further mechanistic studies showed that JWA deficiency blocked TPA-induced activation of MAPKs and its downstream transcription factor Elk1 both *in vitro* and *in vivo*. *JWA* ^Δ*2/*Δ*2*^ mice are resistance to tumorigenesis induced by DMBA/TPA probably through inhibition of transcription factor Elk1 via MAPKs. These results highlight the importance of JWA in skin homeostasis and in the process of skin tumor development.

## Introduction

Viable cells suffer spontaneous DNA damage or genotoxic agent-induced DNA damages. Therefore, a network of DNA surveillance systems has developed in the cells that monitor and coordinate cell cycle progression with repair of damaged DNA to maintain genome integrity. Unrepaired DNA lesions may result in genetic instability, higher frequency of chromosomal aberrations [1], [2] and eventually leading to subsequent tumorigenesis [3], [4].

MAPK (Mitogen-activated protein kinase) pathways are involved in the signal transduction of a wide variety of extracellular stimuli [5], [6], [7]. There are three such classical pathways that activate different MAPK classes, known as ERK (extracellular signal regulated kinase), JNK (Jun N-terminal kinase) and p38, each pathway evokes distinct biological responses. The MEK/ERK pathway is activated by mitogenic stimuli and plays an important role in cell proliferation and differentiation. Activated ERK phosphorylates and activates its targets such as the transcription factor Elk1 (Ets-like transcription factor-1), member of ETS oncogene family. Activated Elk1 organizes ternary complex factor with serum response factor and binds to the serum response element of the promoter of the target genes (e.g. c-fos) and enhances their transcription [8], [9], [10].

The *JWA* gene, also known as *ARL6ip5* (ADP-ribosylation-like factor 6 interacting protein 5), was initially cloned from human tracheal bronchial epithelial cells after treatment with all-trans retinoic acid [11]. Several JWA homologues (e.g. GTRAP3-18, addicsin and JM4) were since identified [12], [13]. Subsequent studies indicated that JWA is involved in the cellular responses to heat shock and chemical-mediated oxidative stresses [14]. JWA plays a key role in protecting cells from DNA damage induced by oxidative stress [15], [16]. On the other hand, there is an increasing amount of data to indicate that JWA is a structurally novel microtubule-associated protein, which regulates cancer cell migration via MAPK cascades [17]. Our recent data have shown that JWA plays an important role in melanoma metastasis via integrin signaling pathway [18]. However, the potential role of JWA in chemically induced skin carcinogenesis has not been elucidated. The purpose of this study was to characterize the role and the related molecular mechanisms of JWA in DMBA-TPA induced two-stage skin papilloma development in conditional JWA knockout mice. Our results demonstrate that JWA deletion enhanced cellular DNA damage induced by DMBA at first stage, nevertheless attenuated tumor incidence induced by TPA at second stage and probably via inactivation of MAPK pathway.

## Materials and Methods

### Generation and genotype identification of JWA mice and cells

**Figure 1:**
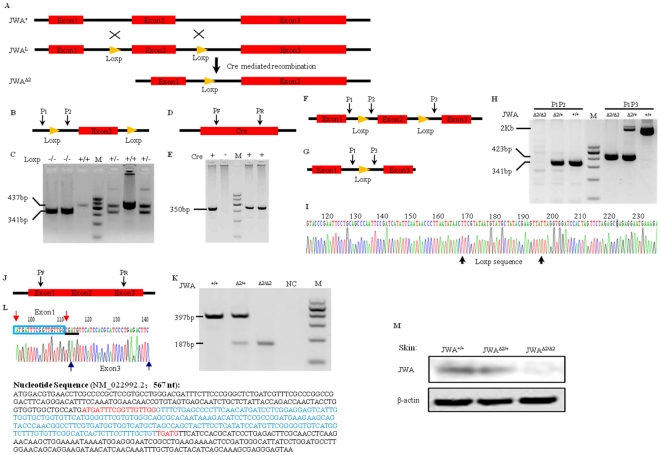
The construction and genotype verification of JWA knockout mice. (A) Schematic representation of JWA gene-targeting strategy. Exon2 of JWA was floxed with Loxp site (*JWA^L^*), after Cre mediated recombination, the exon2 was deleted (*JWA^Δ2^*). (B, C) Genotyping of Loxp mice by PCR. Forward (P1) and reverse (P2) primers used in PCR analysis were shown in (B), the products of PCR acquired were about 437 bp after Loxp inserted. (D, E) Genotyping of *EIIa* Cre mice. (F–I) Genotyping of JWA knockout mice at DNA level. The primers used were shown in (F) and (G). (H) Genotyping of *JWA^Δ2/Δ2^* and *JWA^Δ2/+^* mice by PCR with paired primers. (I) Sequencing result of 341 bp product after exon2 deleted. (J–L) Genotyping of JWA knockout mice at mRNA level. Primers used in RT-PCR analysis were shown in (J). PCR results were shown in (K). The sequencing result of 187 bp product was shown in (L), which is comparable with the Entrze JWA sequence (NM_022992.2). NC, negative control; M, marker. For all the experiments, markers were 600 bp, 500 bp, 400 bp, 300 bp, 200 bp, 100 bp in order. (M) The skin protein from *JWA^+/+^*, *JWA^Δ2/+^* and *JWA^Δ2/Δ2^* mice were analyzed by Western-blot. β-actin was used as the loading control.

The conditional *JWA* ^Δ2/Δ2^ mice were created by contract service at the Model Animal Research Center of Nanjing University, Nanjing, China. Bacterial artificial chromosome-retrieval methods were used for constructing the targeting vector. In brief, an 11 kb genomic DNA fragment containing 5′ element to exon2 of the *JWA* was retrieved from a 129/sv BAC clone bMO 366n04 by a retrieval vector containing 2 homologous arms. Exon2, which encodes the majority of conserved PRA-1 (prenylated rab acceptor) domain, was flanked by 2 *loxP* sites and an *frt-Neo-frt* cassette as a positive selection marker. In theory, this deletion will cause an out-of-frame reading shift and thereby generate a premature stop codon and a loss-of-function allele (Fig. 1). Embryonic stem W4 cells were electroporated with the linearized targeting vector, selected, then expanded for Southern blot analysis. Chimeric mice were generated by injecting ES cells into C57BL/6 blastocysts followed by transferring to pseudopregnant mice. These chimeric mice (*JWA^loxP/+^*) were viable and finally interbred to generate *JWA^loxP/loxP^* mice, which are healthy, fertile and have reached maturity. To generate *JWA* ^Δ2/+^ mice, *JWA^loxP/loxP^* mice were crossed with mice transgenic for *EIIa-Cre* (adenovirus early transcription region IIa promoter-Cre), which express Cre in germ cells. JWA null mutant mice (*JWA* ^Δ2/Δ2^) were produced by intercrossing the *JWA* ^Δ2/+^ mice. Subsequent breeding yielded genotypes for experiments. All experiments were conducted in accordance with Animal Care and Use Committee of Model Animal Research Centre.

For mice and cells genotyping, genomic DNA from the tip of tail of 4-week-old pups or cells was extracted by means of standard protocols. The following primers were used for simultaneous detection of wild-type and null JWA alleles: 5′-CCACTGTTTCCTCTGTTG (P1, forward primer for wild-type and null JWA); 5′-GTGAAAACCACTGAGAACC (P2, reverse primer for wild-type JWA); 5′-CAGATGTTCCTCGTGTATC (P3, reverse primer for null JWA). 5′-ATCAACGTTTTCTTTTCGG (forward primer for *cre*) and 5′-ATTTGCCTGCATTACCGGTC (reverse primer for *cre*).

For mouse and cells genotype confirmation at RNA level, the RNA from mice or cells was transcribed and subjected to RT-PCR analysis by standard protocol. The following primers were used for detection of *JWA* ^+/+^ and *JWA* ^Δ2/Δ2^ mice: 5′-AACCGTGTAGTGAGCAATCTTG (forward primer) and 5′-GGATAATGCCCATCGGAGT (reverse primer). The PCR products from genomic DNA and cDNA were subject to further sequencing analysis for final verification.

### Skin papilloma induction by DMBA/TPA

All the mice used for experiments were maintained in the C57BL/6 background with at least six backcrosses from the original 129Sv/C57BL/6 founder mice. Both wild type and *JWA^Δ2/Δ2^* mice were used in this DMBA/TPA two-stage papilloma induction assay. All the genotypes of mice and cells were verified at genomic DNA and cDNA level, respectively. A total of 48 mice (8–9 weeks old) were divided into 2 groups, each with either the *JWA^+/+^* (n = 24) or *JWA^Δ2/Δ2^* (n = 24) genotype and identical number of male (n = 10) and female (n = 14) mice.

To induce skin papillomas, mice were shaved on their dorsal skin, and 2 days later treated topically with 25 µg of DMBA (Sigma, St. Louis, MO) in 100 µl acetone once. One week later, each animal received subsequent topical treatments of 2.5 µg of TPA (Sigma, St. Louis, MO) in 100 µl acetone twice a week for 19 weeks. Treated mice were examined twice a week for detecting the presence of skin papillomas, which were not scored as positive until they reached at least 1 mm in diameter. At the end of the two-stage model, all mice were sacrificed, and skin papillomas were counted and isolated for further histological analysis. All experiments were conducted in accordance with Animal Care Committee of Nanjing Medical University.

### Histological and immunohistochemical analyses

Dorsal skin and papilloma samples were isolated and fixed in 4% paraformaldehyde at 4°C overnight, embedded in paraffin, and sectioned as 4 µm slides. The sectioned tissues on slides were stained with hematoxylin and eosin. Immunohistochemical staining was further carried out using indicated first antibodies and the Immuno Cruz Staining Systems (Zhongshan Golden Bridge Biotechnology, Beijing, China). The endogenous peroxidase activity in the specimens was blocked by treatment of 0.3% H_2_O_2_ and the samples were then rinsed with PBS. The specimens were probed consecutively with primary antibody against PCNA (1∶100, Epitomics, California, USA), Ki67 (1∶200, Abcam, Cambridge, UK) for 2 h, biotin-conjugated goat anti-rabbit IgG for 30 min, horseradish peroxidase-streptavidin complex, and then developed with diaminobenzidine.

### Preparation of mouse embryonic fibroblast (MEF) and keratinocytes

On day 13.5 of gestation, embryos from *JWA* ^*+/*Δ*2*^×*JWA* ^*+/*Δ2^ crossed females were harvested. Embryo head and all visible organs (heart, spleen, etc.) were removed. Remaining embryo was put in 50 ml tube and minced with scissors. 5 ml 0.25% trypsin was added and incubated in 37°C water bath for 20 min. Trypsin was inactivated using 5 ml of Dulbecco's Modified Eagle Medium (DMEM) containing 10% fetal bovine serum. The harvested cells were centrifuged to pellet at 1500×g for 5 min and then resuspended with 15 ml of fresh medium. Cell suspension was allowed to stand for 5–10 min to let the debris settle to the bottom. Top 10 ml of medium containing cells was removed and plated in a 100-mm dish.

For isolating keratinocytes, full-layer skin removed from newborn (1–2 days old) mice was treated with 0.25% trypsin (Invitrogen) overnight at 4°C. The epidermis was then peeled off from the dermis and minced into pieces smaller than 1 mm, and placed into a sterile flask, dispersed by stirring into single cells for 30–60 min, suspended in keratinocyte-SFM with supplements (Invitrogen). Cells were first incubated in dishes coated with type I collagen at 34°C in 5% CO_2_ for 12 h to allow cells to attach. Unattached cells were removed by washing with PBS. Attached cells were further cultured in fresh medium, which was refreshed every 2 days.

### Neutral comet assay

The keratinocytes were cultured in standard medium for 4 days, and then treated with or without 0.15 µg/ml DMBA for another 24 h. The comet assay was carried out according to the manufacturer's instructions. Briefly, cells at a concentration of 5×10^5^/ml in PBS were mixed gently with pre-melted low-temperature-melting agarose at a volume ratio of 1 to 9 (v/v) and spread on glass slides which were coated with normal-temperature agarose. The slides were then submerged in pre-cooled neutral lysis buffer at 4°C for 90 min. After rinsing, the slides were electrophoresed at constant 25 V, 300 mA for 20 min, then equilibrated in Tris-borate EDTA solution, and stained with ethidium bromide. Fluorescent images for at least 50 nuclei were captured using an Olympus microscope and analyzed by CASP-1.2.2 software for tail moment.

### Immunofluorescence

Cells grown on coverslips for 24 h were treated with or without 0.15 µg/ml DMBA for 24 h. After washing with PBS three times, cells were fixed with methanol for 10 min followed with PBS wash twice, and then incubated in PBS containing 5% BSA for 1 h. After washing with PBS twice, the cells were incubated with anti-phosphorate γ-H2AX primary antibody (1∶300, Upstate, NY) in PBS containing 5% BSA at 4°C overnight. Afterwards, coverslips were washed twice for 5 min in PBS and incubated with Texas Red-conjugated anti-mouse antibody (1∶300 in PBS containing 5% BSA) for 1 h. Finally, coverslips were counterstained with DAPI (10 ng/ml) for 10 min. The images were captured using a fluorescent microscope.

### Quantitative real-time PCR

Total RNA was extracted from cells or tissues with TRIzol (Invitrogen). Total RNA (2 µg) was reverse transcribed with oligo (d18T) primer using the M-MLV reverse transcriptase for RT-PCR. The cDNA was used as template for quantitative real-time PCR analysis, preformed using SYBR Premix Ex Taq Mix (TaKaRa) with ABI Prism 7900 sequence detection system. Reactions were in triplicate for each sample and data were normalized by GAPDH levels. The following primer pairs were used for the PCR reactions: Elk1, forward primer(F): 5′-TTTGTGTCCTACCCAGAGGTT-3′ and reverse primer (R): 5′-CTGCACCCTTTGGTGTTCC-3′; c-fos, F: 5′-CCCATCCTTACGGACTCCC- 3′ and R: 5′-GAGATAGCTGCTCTACTTTGCC-3′; c-myc, F: 5′-TCTCCATCCTATGTTGCGGTC-3′ and R: 5′- TCCAAGTAACTCGGTCATCATCT-3′; PCNA, F: 5′-TGCTCTGAGGTACCTGAACT-3′ and R: 5′- TGCTTCCTCATCTTCAATCT-3′; GAPDH, F: 5′-ACAGCCGCATCTTCTTGTGC-3′ and R: 5′-CACTTTGCCACTGCAAATGG-3′. We used the following PCR procedure: 94°C for 10 min, then 40 cycles of 95°C for 15 s, 60°C for 1 min, 72°C for 45 s.

### Western blot analysis

Papillomas and dorsal skin of the mice were isolated and homogenized with a grinder for 10 min in RIPA lysis buffer as previously described [19]. Cellular protein was extracted by whole cell extract protocols from cell pellets in protein lysis buffer containing protease and phosphatase inhibitors. Western blot analysis was performed by standard procedures [18]. Membranes were incubated with antibodies detecting phosphorylated B-Raf (Ser 445), MEK (Ser 217/221), ERK1/2 (Thr202/Tyr204), JNK (Thr183/Tyr185), p38 (Thr180/Tyr182), total B-Raf, MEK, ERK1/2 , JNK, p38 and α-tubulin (1∶1000, all from Cell Signaling Technology, Inc. Danvers, MA); JWA (1∶1000, Imgenex, San Diego, CA, USA), Elk1, c-fos, c-myc (1∶1000, Bioworld, Atlanta, Georgia); PCNA (1∶2000, Epitomics, California, USA), β-actin (1/1000, Boster Technology, Wuhan, China)

### Statistical analysis

Data were analyzed by Prism software 5.0 (GraphPad Software Inc., La Jolla, CA). The Kaplan-Meier method was used for comparison of the tumor development induced by DMBA/TPA. The Student's *t*-test was performed to determine statistical significance for neutral comet assay and relative expression levels. Wilcoxon rank-sum test was used to compare the difference in tumor numbers. In all the above analyses, a *P* value of <0.05 was considered statistically significant.

## Results

### Targeted disruption of the mouse JWA gene

To investigate the role of JWA in the development of mammalian skin tumors, we constructed the conditional JWA knockout mice. Exon2 of JWA was floxed with Loxp site (*JWA^L^*), after Cre mediated recombination, the exon2 was deleted (*JWA* ^Δ*2*^) (Fig. 1A). The conditional JWA knockout mice (*JWA* ^Δ2/Δ2^) were produced by intercrossing the *JWA* ^Δ2/+^ mice. Genotyping of Loxp was shown in Fig. 1B (PCR primers location) and Fig. 1C (LoxP marker was shown in lanes contain 437 bp) and genotyping of *EIIa* Cre was indicated in Fig. 1D–E. Genotyping of JWA knockout mice were identified at genome DNA level (Fig. 1F–G for primers location; Fig. 1H for PCR products: non product for P1–P2 amplification in *JWA* ^Δ2/Δ2^, 341 bp for *JWA* ^Δ2/+^and *JWA^+/+^*, 423 bp for *JWA* ^Δ2/+^and *JWA* ^Δ2/Δ2^ in P1–P3 products; and sequncing confirmed in Fig. 1I); mRNA level (Fig. 1J–L) and protein level (Fig. 1M). Although the *JWA^Δ2/Δ2^* mice developed premature ageing like phenotypes such as decreased body weight, kyphosis, osteoporosis, and immune organ atrophy, we have not found any spontaneous tumors during their lifespan (described elsewhere).

### JWA deficiency attenuates the development of mouse skin papillomas

**Figure 2:**
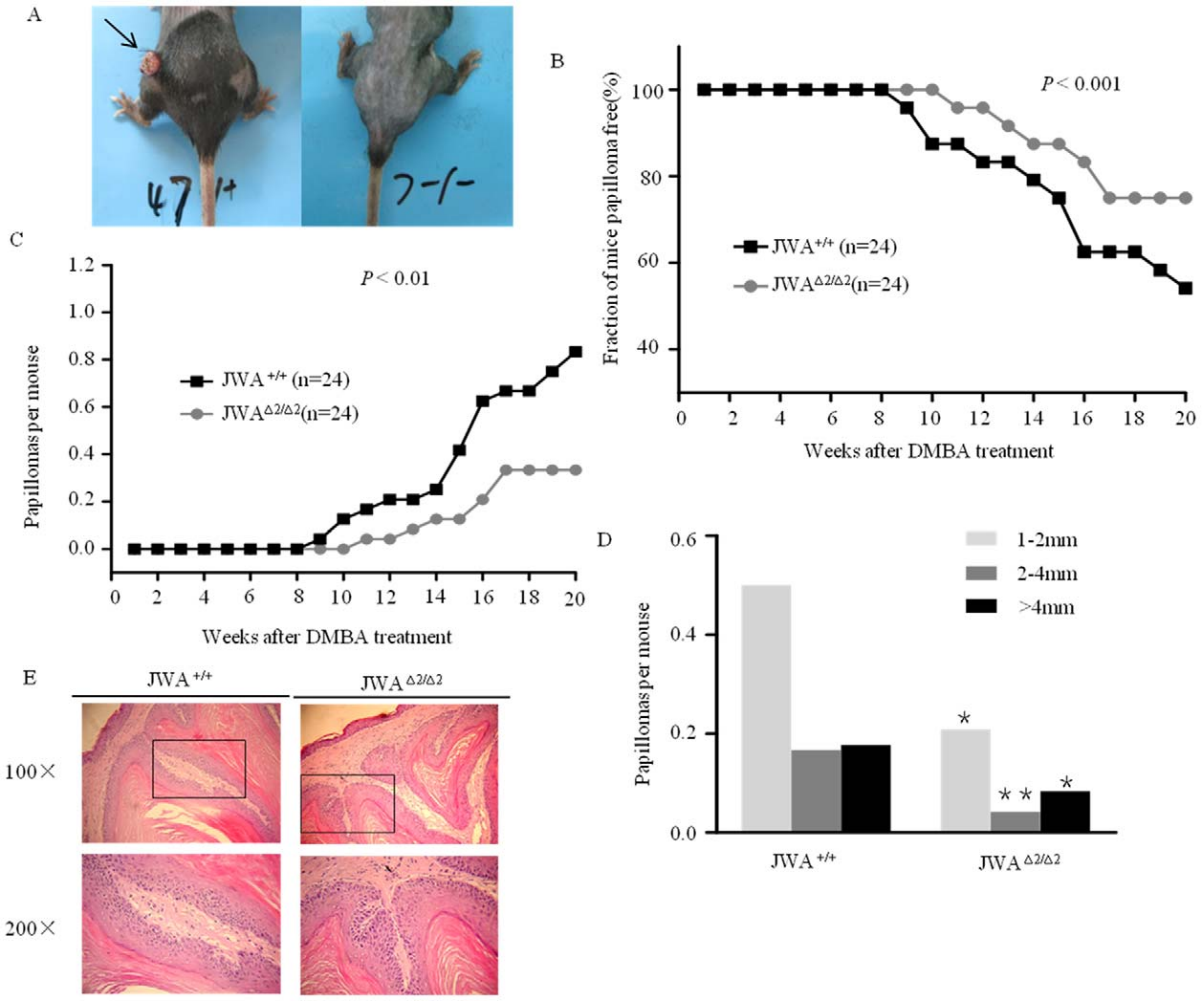
Mouse skin papillomas induced by DMBA/TPA. (A) Papillomas induced by DMBA/TPA treatment in *JWA^+/+^* mouse skin (left). Arrow indicates the papilloma. (B) Comparison of the percentage of papilloma-free mice between *JWA^+/+^* and *JWA* ^Δ*2/*Δ*2*^ groups after DMBA/TPA treatment. ***P<0.001. (C) Comparison of average numbers of papillomas per mouse between *JWA^+/+^* and *JWA* ^Δ*2/*Δ*2*^ groups. ***P<0.001. (D) Comparison of papilloma size distribution between *JWA^+/+^* and *JWA* ^Δ*2/*Δ*2*^ groups. The diameter of papillomas was used to represent the tumor size. *P<0.05, **P<0.01. (E) H&E staining for typical papillomas from both *JWA^+/+^* and *JWA* ^Δ*2/*Δ*2*^ mice.

As shown in Fig. 2A, skin papilloma was induced by DMBA/TPA treatment in both *JWA^+/+^* and *JWA* ^Δ*2/*Δ*2*^ mouse skin. In the present study, the first papilloma was observed after 8 weeks of TPA treatment in *JWA^+/+^* mouse and appeared 2 weeks later in *JWA* ^Δ*2/*Δ*2*^ mouse than in *JWA^+/+^* mouse (Fig. 2B). At the 19^th^ week of TPA treatment, the end point of experiment, 11 *JWA^+/+^* mice and 6 *JWA* ^Δ*2/*Δ*2*^ mice developed skin papillomas. The ratio of tumor induction in *JWA^+/+^* mice was significantly higher than in *JWA* ^Δ*2/*Δ*2*^ mice (*P*<0.001). There were significantly fewer number and smaller sizes of papillomas occurred in *JWA* ^Δ*2/*Δ*2*^ mice than in *JWA^+/+^* mice (*P*<0.05 and *P*<0.01, respectively; Fig. 2 C and D). Skin specimens with H&E staining confirmed that the papillomas with no significant difference between the mice (Fig. 2E). These data suggest that JWA deficiency attenuates the initiation and development of mouse skin papillomas induced by DMBA/TPA treatment.

### JWA deficiency inhibits DMBA/TPA induced cell proliferation

**Figure 3:**
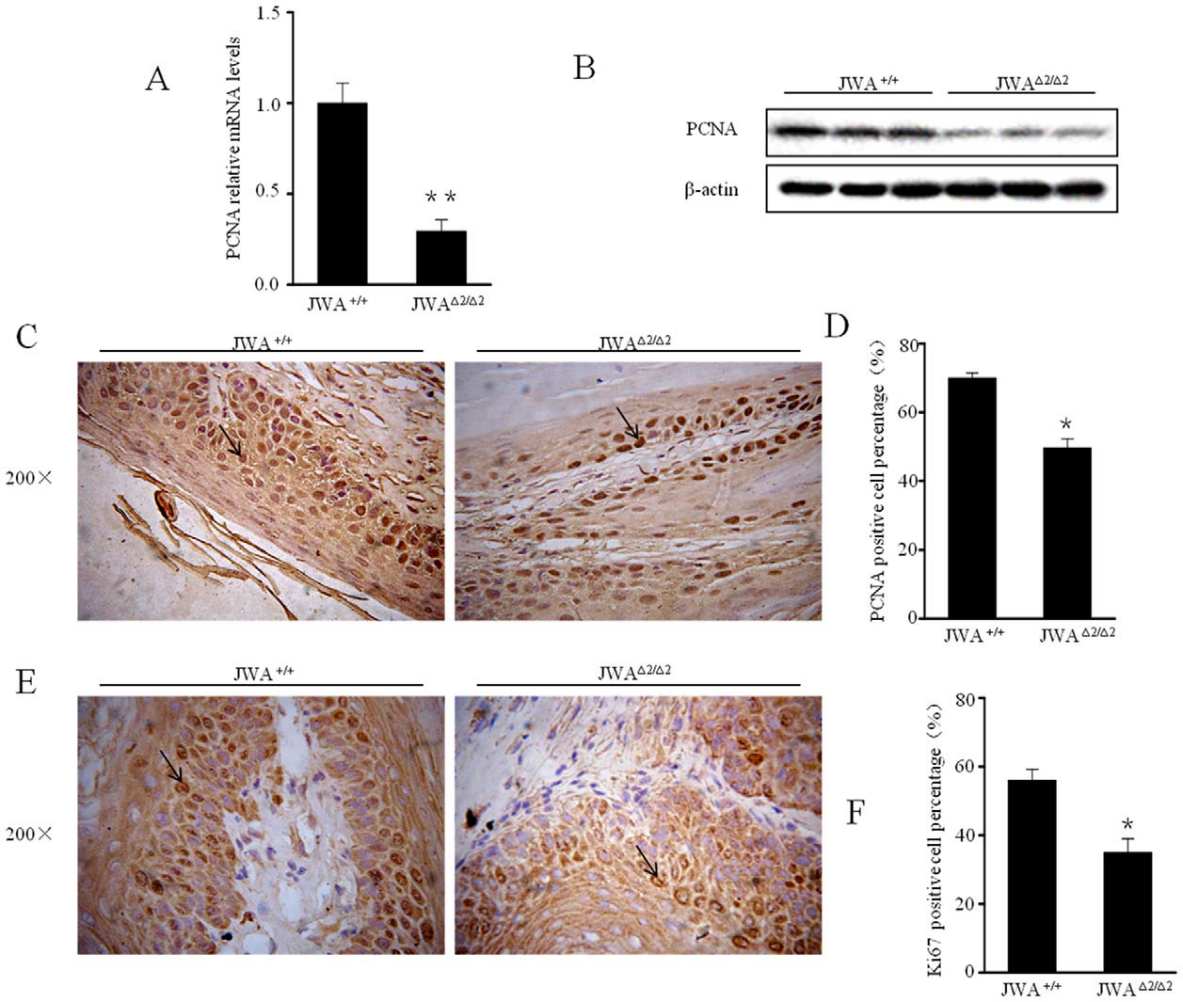
The expression of PCNA and Ki67 in mouse papillomas treated with DMBA/TPA. PCNA expression in the papillomas of *JWA^+/+^* and *JWA* ^Δ*2/*Δ*2*^ mice (n = 3) treated with DMBA/TPA was analyzed at mRNA level by real-time PCR (A) and protein level by Western blotting (B). * *P*<0.05. (C, E) Typical PCNA and Ki67 immunostaining in papillomas from *JWA^+/+^* and *JWA* ^Δ*2/*Δ*2*^ mice. Arrows indicate PCNA and Ki67 positive cells. (D, F) The numbers of PCNA and Ki67 positive epidermal cells were counted from at least 100 cells in five separate fields for each section respectively (n = 3). *P<0.05. Data were presented as means ± s.d. from three independent experiments.

To understand whether reduced papillomas in JWA knockout mice was due to inhibition of cell proliferation, expression of PCNA in mouse papillomas and skin tissues nearby was analyzed. As we predicted, data showed the expressions of mRNA (*P*<0.01) (Fig. 3A), protein (Fig. 3B), and the amount of PCNA-positive cells in papillomas was significantly higher in *JWA^+/+^* than in *JWA* ^Δ*2/*Δ*2*^ mice (*P*<0.05) (Fig. 3C and D). Similar result was obtained from the expression of Ki67 in mouse papillomas (Fig. 3E and F). In addition, the expression levels of PCNA between mouse skin tissues and the papillomas have shown no difference (Fig. S1).

### JWA deficiency blocks TPA-mediated phosphorylations of MAPKs

**Figure 4:**
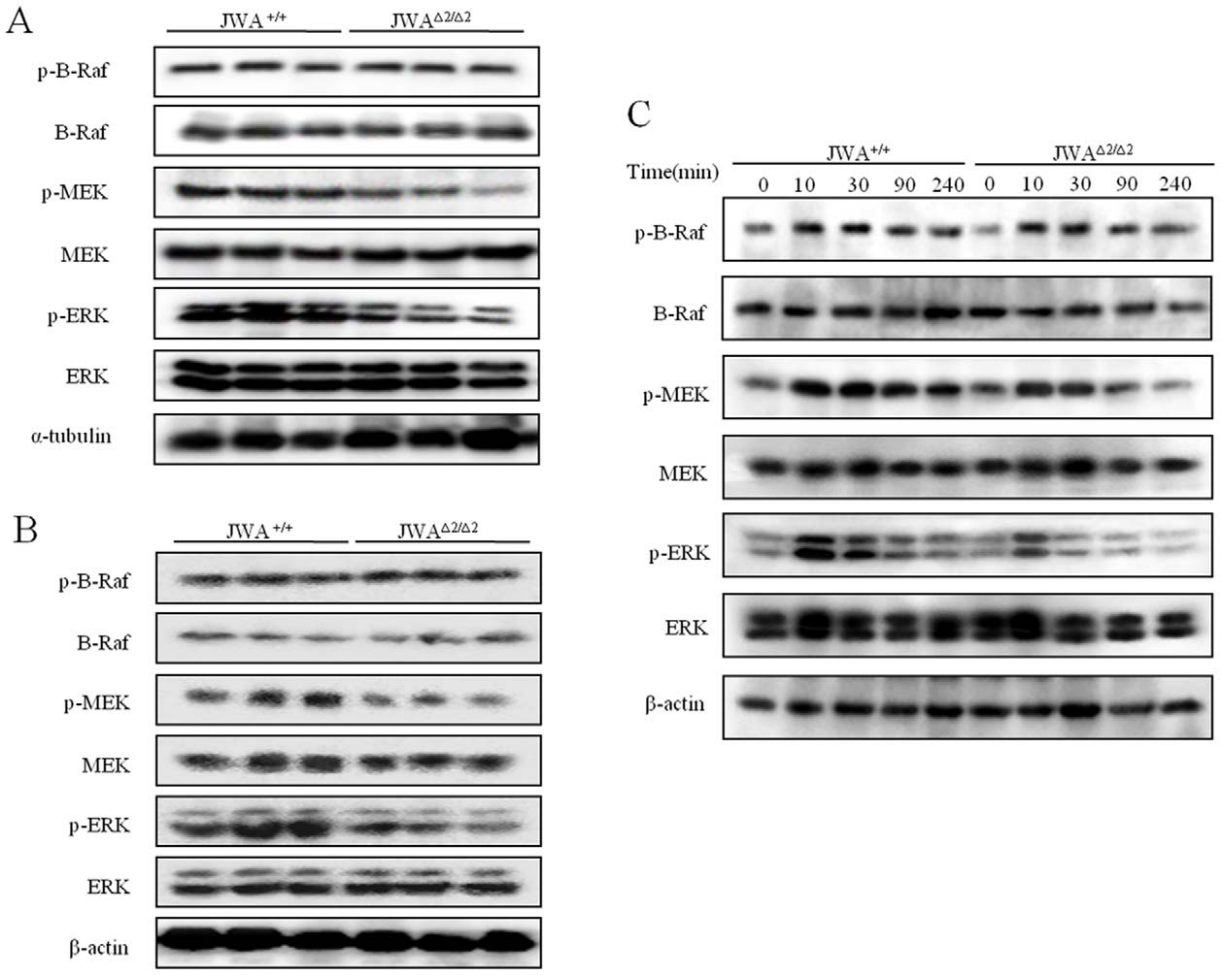
TPA-stimulated phosphorylations of MAPKs in *JWA^+/+^* and *JWA* ^Δ***2/***Δ***2***^ **mouse papillomas, skin tissue and keratinocytes.** (A–B) Epidermal lysates were prepared in tissue protein extraction buffer from *JWA^+/+^* and *JWA* ^Δ*2/*Δ*2*^ mouse papillomas (A) and skin (B) treated with DMBA/TPA at the end point of experiment. Total protein (60 µg per well) from paired samples (n = 3) was run on SDS-PAGE and probed with antibodies for phosphorylated or total amounts of B-Raf, MEK and ERK. (C) *JWA^+/+^* and *JWA* ^Δ*2/*Δ*2*^ keratinocytes were treated with 100 ng/ml TPA at indicated time points. Phosphorylations or total amounts of B-Raf, MEK and ERK were determined by Western blotting. β-actin was used as the loading control. Each experiment was performed in triplicate.

Cellular proliferation can be mediated by the activation of MAPK signal pathway. We previously reported that JWA as critical activator of MAPK signal pathway involves in the regulation of cell migration [17]. ERK activity was essential for the development of skin papillomas induced by the classic DMBA/TPA skin carcinogenesis protocol [19], [20]. To determine whether JWA deficiency attenuated papilloma formation was due to inactivation of MAPKs in mice; both papillomas and skin tissues nearby were extracted for Western blot analysis. As a result, compared to the *JWA* ^+/+^ mice, less activation of p-MEK and p-ERK in *JWA* ^Δ*2/*Δ*2*^ mice was found (Fig. 4A and B), although the total expressions of MEK and ERK were unaffected. Interestingly, both phosphorylation and expression of JNK and p38 proteins were also unaffected in papillomas of both *JWA* ^+/+^ and *JWA* ^Δ*2/*Δ*2*^ mice (Fig. S2A).

The primary keratinocytes of the both genotypes mice were isolated to verify if JWA deletion blocks the role of TPA on the activation of MAPKs. As shown in Fig. 4C, TPA treatment resulted in more intensive phosphorylations of MEK and ERK in *JWA^+/+^* keratinocytes, however, this effect was obviously reduced and did not last in *JWA* ^Δ*2/*Δ*2*^ keratinocytes. Furthermore, our data also confirmed *in vivo* result that TPA had no effect on JNK and p38 proteins in both *JWA^+/+^* and *JWA* ^Δ*2/*Δ*2*^ keratinocytes (Fig. S2B).

### JWA regulates transcription factor Elk1 via MEK/ERK pathway

**Figure 5:**
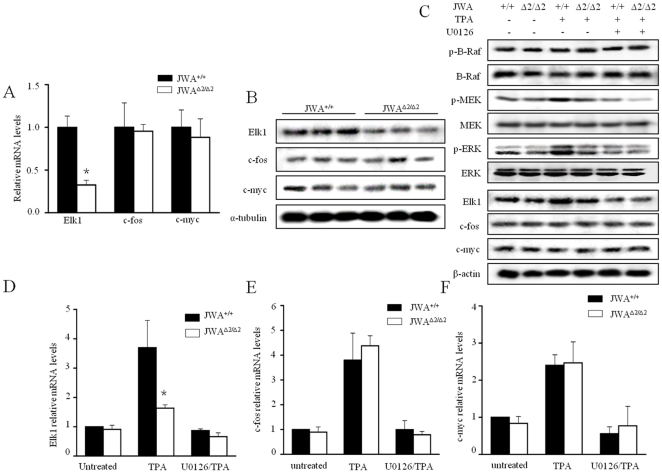
Transcription factor Elk1 was regulated by JWA. (A–B) Elk1, c-fos and c-myc expression in the papillomas from *JWA^+/+^* and *JWA* ^Δ*2/*Δ*2*^ mice (n = 3) were analyzed at mRNA level by real-time PCR (A) and at protein level by Western blotting (B). (C) *JWA^+/+^* and *JWA^Δ2/Δ2^* keratinocytes were treated with or without 100 ng/ml TPA for 30 min or with 5 µg/ml U0126 (MEK inhibitor) for 6 h to silent MEK/ERK signaling, and then treated with 100 ng/ml TPA for 30 min. Related protein expressions were shown. (D–F) The mRNA expression of Elk1 (D), c-fos (E) and c-myc (F) in *JWA^+/+^* and *JWA* ^Δ*2/*Δ*2*^ keratinocytes were analyzed by real-time PCR after TPA and/or U0126 treatment. *P<0.05. Data were presented as means ± s.d. from three independent experiments.

It has been reported that transcription factors Elk1, c-fos and c-myc are all highly related to cell proliferation, and regulated by MEK/ERK pathway [21], [22]. We investigated if the role of JWA on PCNA was mediated by any of these transcription factors. As a result, compared to *JWA^+/+^*mice, only expressions of Elk1 at both mRNA (*P*<0.05) (Fig. 5A and Fig. S3A) and protein (Fig. 5B and Fig. S3B) levels were significantly down-regulated in *JWA^Δ2/Δ2^* mouse papillomas and skin tissues.

To investigate if TPA treatment would affect Elk1 expression via activation of MAPKs, we treated *JWA^+/+^* and *JWA* ^Δ*2/*Δ*2*^ keratinocytes with TPA and found that Elk1 expression was only increased in *JWA^+/+^* keratinocytes (Fig. 5C). There was no significant difference in protein level of c-fos and c-myc in keratinocytes of both genotypes after treatment with TPA alone or with the MEK inhibitor U0126. Similarly, TPA induced Elk1 mRNA expression (*P*<0.05), and no effects on c-fos and c-myc (Fig. 5D–F). Similar results were obtained from MEFs (Fig. S3C–F). These data provide further evidence that JWA may regulate Elk1 transcription factor via MEK/ERK pathway.

### JWA deficiency enhances DMBA-induced DNA damage

MAPK pathway was shown to be involved in DNA damage repair process [23], [24]. To investigate if MAPK pathway is involved in DMBA-induced DNA damage repair, phosphorylations of MEK/ERK were examined and results indicated that DMBA does not cause significant change on MEK/ERK phosphorylation (Fig. S4).

**Figure 6:**
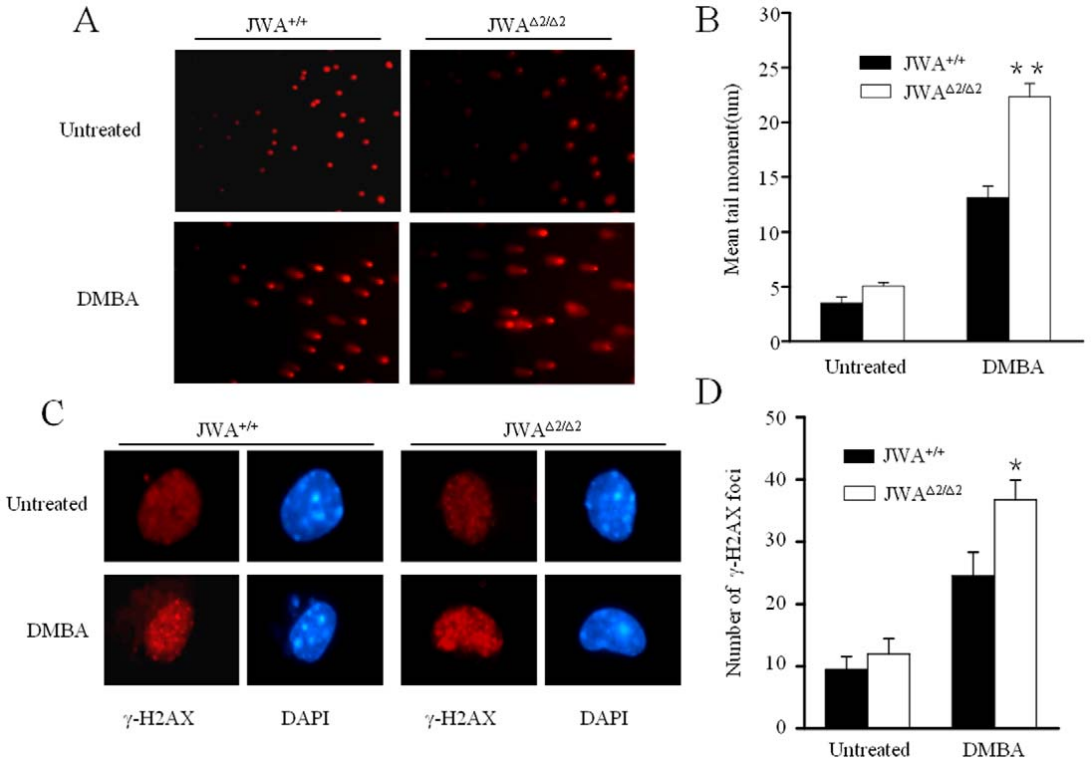
JWA deficiency enhances the DMBA-induced DNA damage. (A) DNA strand breaks in *JWA^+/+^* and *JWA* ^Δ*2/*Δ*2*^ keratinocytes after DMBA treatment. Cells were either treated with or without 0.15 µg/ml DMBA for 24 h, and DNA strand breaks were detected by neutral comet assay. (B) Quantification of comet tail movement in (A) from 50 cells in each sample in three independent experiments. **P<0.01. (C) γ-H2AX staining in *JWA^+/+^* and *JWA* ^Δ*2/*Δ*2*^ keratinocytes after DMBA treatment. Cells were treated with or without 0.15 µg/ml DMBA for 24 h, and DNA strand breaks were detected with γ-H2AX labeling. (D) Quantification of γ-H2AX foci. Foci were counted in at least 50 cells for each sample, and the results were presented as the mean ± s.d. * *P*<0.05.

Since JWA is a DNA damage repair associated protein, we further investigated if JWA deficiency enhances DMBA-induced DNA damage and genome instability. The neutral comet assay was used to detect DNA double strand breaks (DSBs) of keratinocytes induced by DMBA treatment. As a result, DMBA induced more DSBs in *JWA* ^Δ*2/*Δ*2*^ keratinocytes than in *JWA^+/+^* cells (*P*<0.01) (Fig. 6A and B). Consistent with the result from neutral comet assay, *JWA* ^Δ*2/*Δ*2*^ keratinocytes had more γ-H2AX positive foci than in *JWA^+/+^* cells after DMBA exposure (*P*<0.05) (Fig. 6C and D).

## Discussion

*JWA* was initially isolated as an all-trans-retinoic acid responsive and cytoskeleton-associated gene [11]. Previously, we identified JWA as a novel mitogen activated protein, which binds to α- and β-tubulin and is essential for the rearrangement of F-actin cytoskeleton and activation of MAPK cascades induced by As_2_O_3_ and TPA [17]. Down-regulation of JWA accelerates melanoma cell migration and adhesion, and promotes cell invasion through matrigel-coated chamber *in vitro* [18]. On the other hand, JWA was regulated by environmental stressors such as heat shock and oxidative stress [14], [25]. JWA also participated in the protection of cells from oxidative stress-induced DNA damage [15]. Therefore, JWA is precisely involved in both DNA damage repair process and regulation of MAPK pathway. In the present study, we examined whether combined treatment with DMBA and TPA will affect the development of skin papillomas in *JWA* ^Δ*2/*Δ*2*^ mice. The data showed that although JWA deficiency enhanced DMBA-induced DNA damage *in vitro*, TPA promotion on the development of skin papillomas was reduced in *JWA* ^Δ*2/*Δ*2*^ mice compared with *JWA^+/+^* mice. These results verified the unique role of MEK/ERK in TPA tumor promotion model and also indicated for the first time that JWA controls the process as an important checkpoint.

It has been understood for many years that skin carcinogenesis is a multistep process of several separable genetic alterations [26]. The results of mouse skin chemical carcinogenesis studies have provided a conceptual framework for malignant progression. The oxidation of DMBA by P450 enzymes produces metabolites that form covalent adducts with DNA and the formation within DNA of depurinated abasic sites and induces genetic alteration whereby normal cells are converted to a premalignant state. Several DNA repair genes are involved in this step, such as Sirt1, Rad1, Rad9, etc. Rad1 and Rad9 are important for preventing tumor development, probably through maintaining genomic integrity [27], [28], while Sirt1 does not behave like a classical tumor-suppressor but partly participates the antitumor activity of resveratrol [29]. Our present study suggests that JWA, as a DNA repair protein, actively responds to DMBA-induced cell stress, which is consistent with the previous studies [15], [16]. JWA deficiency cells acquired more severe injury than JWA wild type cells, suggesting JWA deletion increased the susceptibility to tumor induction might be due to reduced DNA repair capacity of cells. This seems to be contradictory to the results from skin carcinogenesis animal model showing that JWA deficiency attenuated tumor formation induced by DMBA/TPA treatment. However, further evidences indicate that JWA deficiency effectively inhibits TPA promotion step. TPA is considered to promote DNA damaged cells to enter hyper-proliferative state via activating transcriptional factors. This step results in clonal expansion of cells to form benign premalignant lesions called papillomas. This promotion step is always involved in the activation of B-Raf/MEK/ERK pathway [19], [30], since this pathway is identified to regulate fundamental processes in normal and malignant cells, including proliferation and cell survival [31]. In response to tumor promotion induced by TPA, the inhibition of MEK/ERK is beneficial because it prevents skin tumor development [20], [32], [33]. We have previously shown that JWA is an upstream activator of MEK. Further analysis in JWA knockout keratinocytes and MEFs revealed that JWA is required for TPA-induced MEK and ERK phosphorylation. Consistent with this result, there was a remarkable decrease in the expression of PCNA in *JWA^Δ2/Δ2^* mice. Therefore, our data indicated that in DMBA/TPA two-stage skin papilloma induction model, MEK-ERK activation is critical for the second step. Without effective activation of MEK-ERK, the cells are less prone to papilloma development even they receive severe DNA damage.

AP-1, which consists of a family of Jun/Fos dimers, is a major positive regulator of cell proliferation and transformation and can be activated by TPA in the skin [34], [35], [36]. In this study, however, we did not find any positive role of AP-1 during DMBA/TPA papilloma induction. Elk1, as a member of ETS oncogene family, is an identified target of MAPK which has been reported to play a key role in cell proliferation [21], [37], [38]. We found reduced Elk1 expression in *JWA* ^Δ*2/*Δ*2*^ mouse skin tissues as well as in cultured *JWA* ^Δ*2/*Δ*2*^ MEFs, suggesting that Elk1 may be involved in TPA induced cell proliferation.

The other two MAP kinase pathways (JNK and p38) have observed to counteract malignant transformation under the stress response [31]. Consistent with those findings, we observed that, unlike the B-Raf/MEK/ERK proteins, TPA treatment increased phosphorylations of neither p38 nor JNK in both skin tissue and keratinocytes.

In this study, the reduced skin tumor formation observed in the *JWA* ^Δ2/Δ2^ mice might be also partially contributed by the similar mechanisms of premature ageing like phenotype, however, the exact molecular evidences need to be further provided. To exclude the contribution of non cell autonomous effects in skin tumor phenotypes observed in the full knockout mice, future studies should include the conditional deletion of this gene from the skin.

In summary, we demonstrate for the first time that JWA deficiency enhances DNA damage in epidermal cells induced by DMBA, however, suppresses TPA-induced MEK-ERK activation, cell proliferation, and formation of skin papillomas. These data has potential clinical implications for targeting JWA in chemoprevention and therapy of skin tumors.

## Supporting Information

Figure S1 **The expression of PCNA in mouse skin treated with DMBA/TPA.** PCNA expression in the skin of *JWA^+/+^* and *JWA* ^Δ*2/*Δ*2*^ mice (n = 3) treated with DMBA/TPA was analyzed at mRNA level by real-time PCR (A) and protein level by Western blotting (B). * *P*<0.05. (C) Typical PCNA immunostaining in skin from *JWA^+/+^* and *JWA* ^Δ*2/*Δ*2*^ mice. Arrows indicate PCNA-positive cells. (D) The numbers of PCNA positive epidermal cells were counted from at least 100 cells in five separate fields for each section (n = 3). *P<0.05. Data were presented as means ± s.d. from three independent experiments.
(TIF)

Figure S2 **TPA-stimulated phosphorylations of MAPKs in** ***JWA^+/+^*** **and** ***JWA^Δ2/Δ2^*** **mouse skin and keratinocytes.** (A) Skin lysates were prepared in tissue protein extraction buffer from *JWA^+/+^* and *JWA* ^Δ*2/*Δ*2*^ mouse skin treated with DMBA/TPA at the end point of experiment. Total protein (60 µg per well) from paired samples (n = 3) was run on SDS-PAGE and probed with antibodies for phosphorylated or total amounts of JNK and p38. (B) *JWA^+/+^* and *JWA* ^Δ*2/*Δ*2*^ keratinocytes were treated with 100 ng/ml TPA for the time period indicated, and phosphorylated or total amounts of JNK and p38 were detected by Western blotting. Each experiment was performed in triplicate.
(TIF)

Figure S3 **Transcription factor Elk1 was regulated by JWA.** (A, B) Elk1, c-fos and c-myc expression in the skin of *JWA^+/+^* and *JWA* ^Δ*2/*Δ*2*^ mice (n = 3) were analyzed at mRNA level by real-time PCR (A) and at protein level by Western blotting (B). (C) *JWA^+/+^* and *JWA* ^Δ*2/*Δ*2*^ MEFs were treated with or without 100 ng/ml TPA for 30 min or with 5 µg/ml U0126 (MEK inhibitor) for 6 h to silent MEK/ERK signaling, and then treated with 100 ng/ml TPA for 30 min. Related protein expressions were shown. (D–F) mRNA expression of Elk1 (D), c-fos (E) and c-myc (F) in *JWA^+/+^* and *JWA* ^Δ*2/*Δ*2*^ MEFs were analyzed by real-time PCR after TPA and/or U0126 treatment. * *P*<0.05. Data were presented as means ± s.d. from three independent experiments.
(TIF)

Figure S4 **The effect of DMBA on phosphorylation of MEK and ERK.** *JWA^+/+^* and *JWA* ^Δ*2/*Δ*2*^ keratinocytes were treated with or without 100 ng/ml DMBA for 30 min or with 5 µg/ml U0126 for 6 h, then by 100 ng/ml DMBA for further 30 min. Phosphorylated or total MEK, ERK expression was determined by Western blotting. β-actin was used for loading control.
(TIF)
