# Supplementary figures and images for: JWA Deficiency Suppresses Dimethylbenz[a]Anthracene-Phorbol Ester Induced Skin Papillomas via Inactivation of MAPK Pathway in Mice
Source: PLoS One. 2012 Mar 26;7(3):e34154. doi: 10.1371/journal.pone.0034154 (PMC3312911; doi:10.1371/journal.pone.0034154)

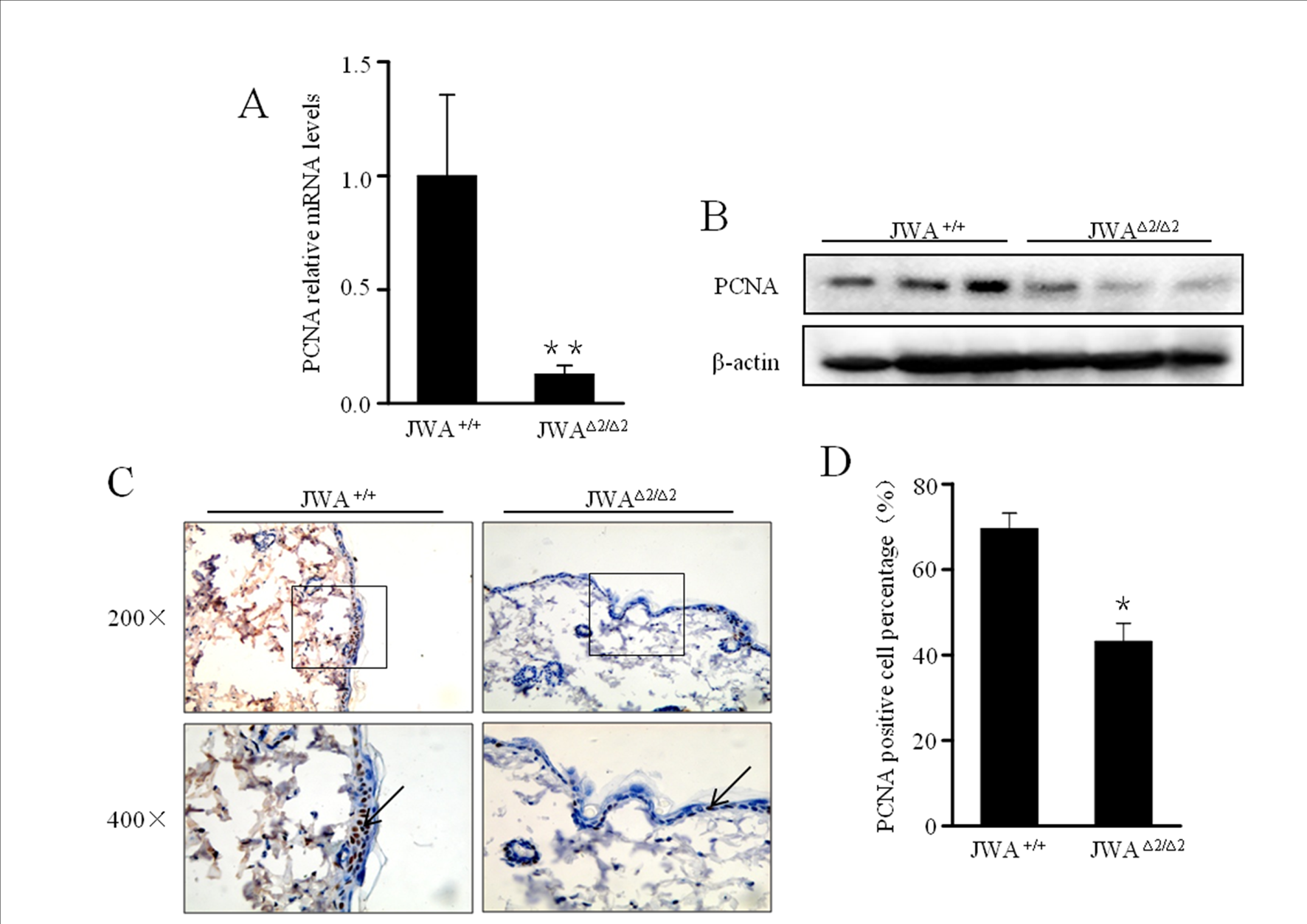

Supplement: Figure S1 — The expression of PCNA in mouse skin treated with DMBA/TPA. PCNA expression in the skin of JWA+/+ and JWA Δ2/Δ2 mice (n = 3) treated with DMBA/TPA was analyzed at mRNA level by real-time PCR (A) and protein level by Western blotting (B). * P<0.05. (C) Typical PCNA immunostaining in skin from JWA+/+ and JWA Δ2/Δ2 mice. Arrows indicate PCNA-positive cells. (D) The numbers of PCNA positive epidermal cells were counted from at least 100 cells in five separate fields for each section (n = 3). *P<0.05. Data were presented as means ± s.d. from three independent experiments. (TIF) [file pone.0034154.s001.tif]

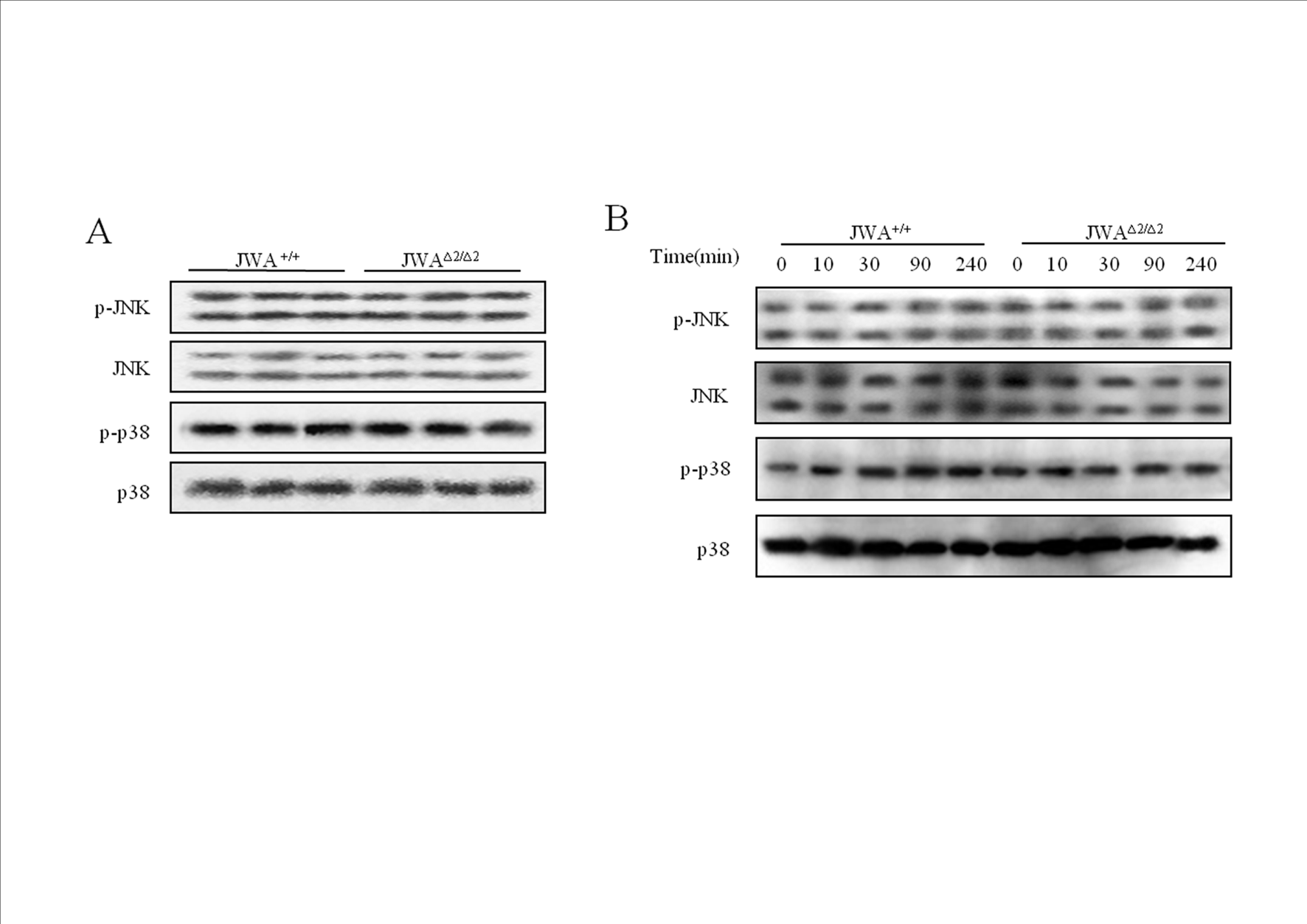

Supplement: Figure S2 — TPA-stimulated phosphorylations of MAPKs in JWA+/+ and JWAΔ2/Δ2 mouse skin and keratinocytes. (A) Skin lysates were prepared in tissue protein extraction buffer from JWA+/+ and JWA Δ2/Δ2 mouse skin treated with DMBA/TPA at the end point of experiment. Total protein (60 µg per well) from paired samples (n = 3) was run on SDS-PAGE and probed with antibodies for phosphorylated or total amounts of JNK and p38. (B) JWA+/+ and JWA Δ2/Δ2 keratinocytes were treated with 100 ng/ml TPA for the time period indicated, and phosphorylated or total amounts of JNK and p38 were detected by Western blotting. Each experiment was performed in triplicate. (TIF) [file pone.0034154.s002.tif]

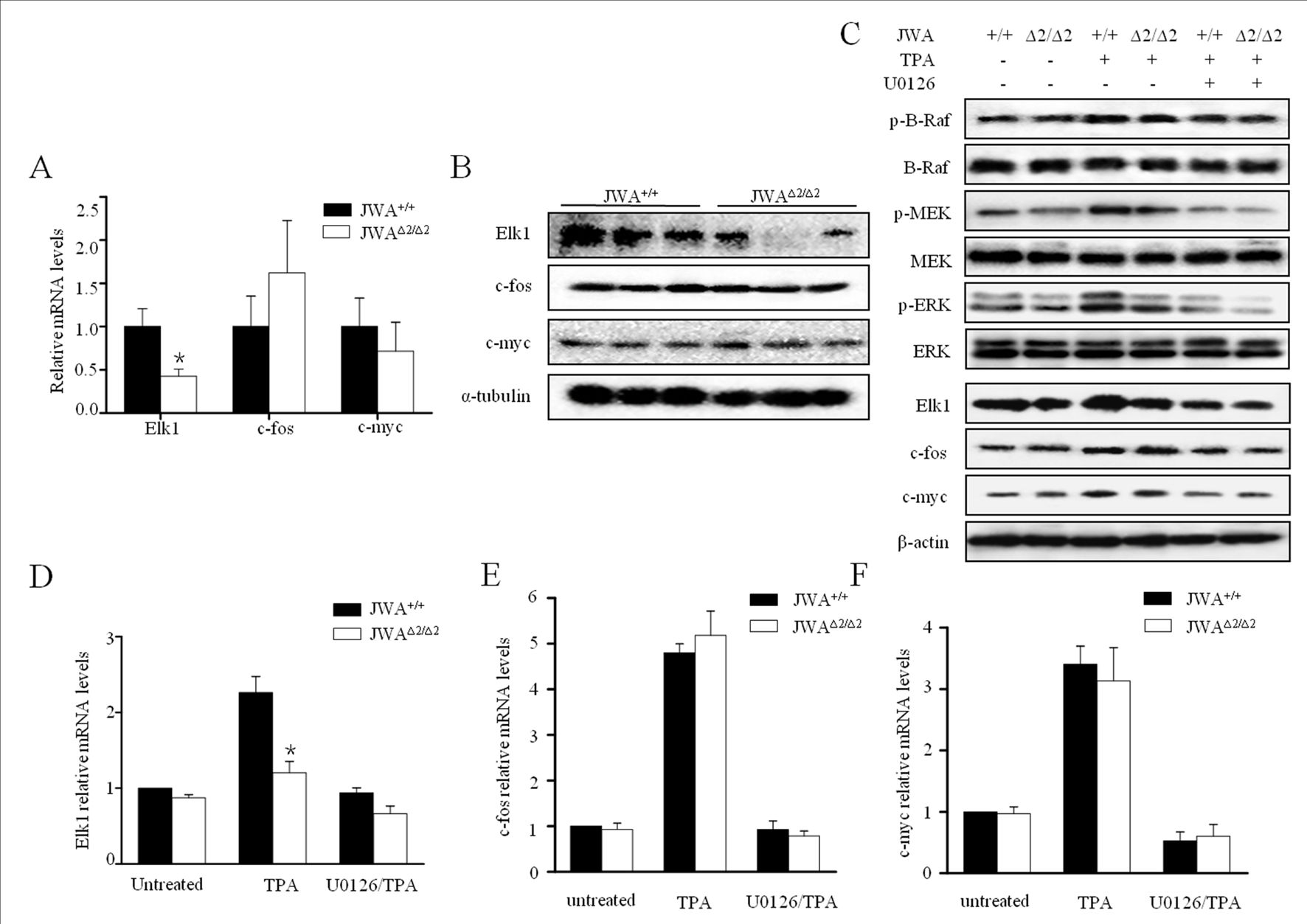

Supplement: Figure S3 — Transcription factor Elk1 was regulated by JWA. (A, B) Elk1, c-fos and c-myc expression in the skin of JWA+/+ and JWA Δ2/Δ2 mice (n = 3) were analyzed at mRNA level by real-time PCR (A) and at protein level by Western blotting (B). (C) JWA+/+ and JWA Δ2/Δ2 MEFs were treated with or without 100 ng/ml TPA for 30 min or with 5 µg/ml U0126 (MEK inhibitor) for 6 h to silent MEK/ERK signaling, and then treated with 100 ng/ml TPA for 30 min. Related protein expressions were shown. (D–F) mRNA expression of Elk1 (D), c-fos (E) and c-myc (F) in JWA+/+ and JWA Δ2/Δ2 MEFs were analyzed by real-time PCR after TPA and/or U0126 treatment. * P<0.05. Data were presented as means ± s.d. from three independent experiments. (TIF) [file pone.0034154.s003.tif]

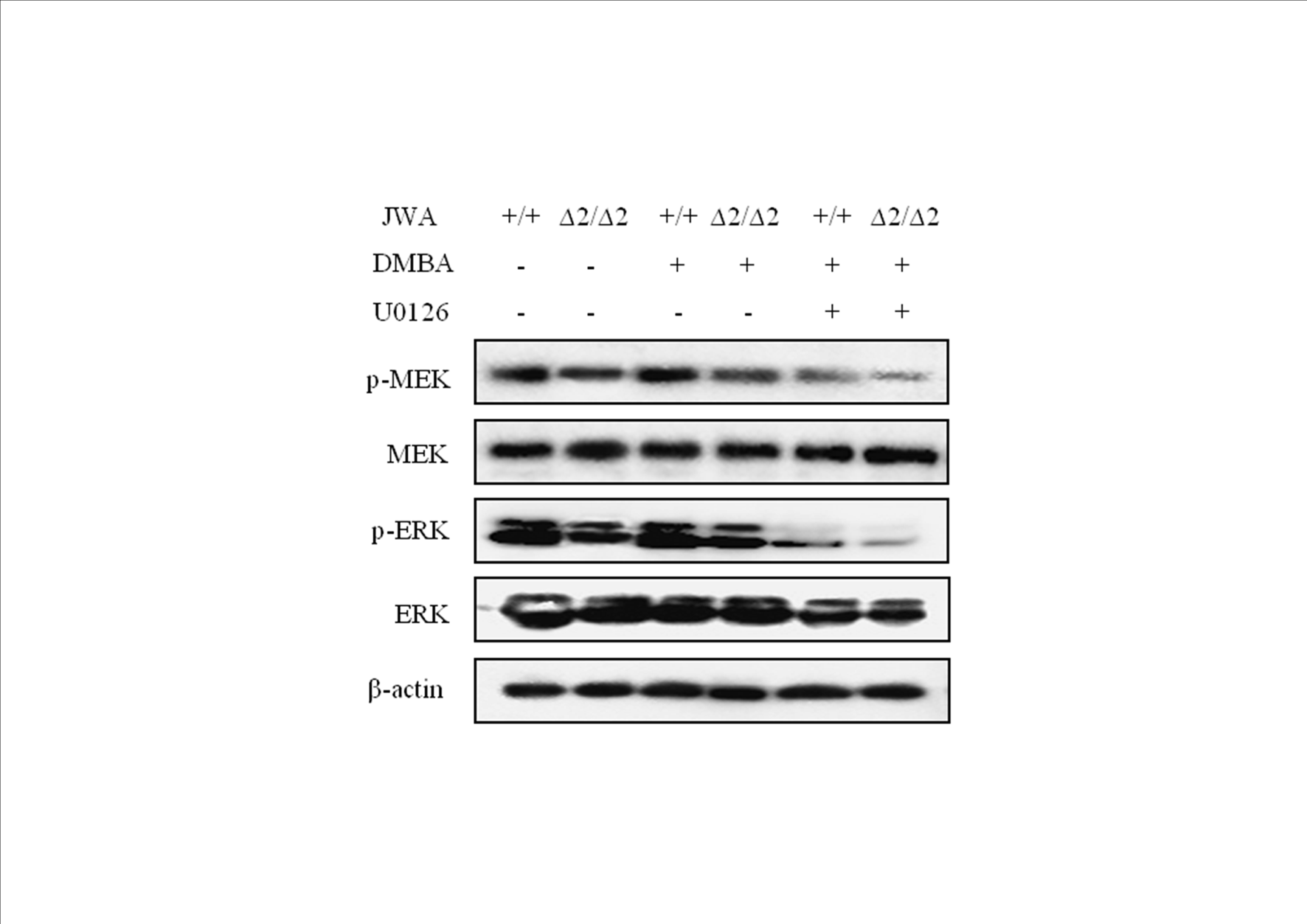

Supplement: Figure S4 — The effect of DMBA on phosphorylation of MEK and ERK. JWA+/+ and JWA Δ2/Δ2 keratinocytes were treated with or without 100 ng/ml DMBA for 30 min or with 5 µg/ml U0126 for 6 h, then by 100 ng/ml DMBA for further 30 min. Phosphorylated or total MEK, ERK expression was determined by Western blotting. β-actin was used for loading control. (TIF) [file pone.0034154.s004.tif]
